# MUSCLE MASS NOT LEAN MASS IS ASSOCIATED WITH HEALTH-RELATED OUTCOMES: IMPLICATIONS FOR DEFINING SARCOPENIA

**DOI:** 10.1093/geroni/igae098.1956

**Published:** 2024-12-31

**Authors:** Peggy Cawthon

**Affiliations:** California Pacific Medical Center, San Francisco, California, United States

## Abstract

The Osteoporotic Fractures in Men (MrOS) Study is the largest study to date that has employed the D3Cr dilution method to assess muscle mass. In the study of ~1400 older men, low D3Cr muscle mass (but not low DXA lean mass or appendicular lean mass) was associated cross-sectionally with worse Short Physical Performance Battery (SPPB) score, slower walking speed, decreased lower extremity power, worse chair stand ability, and greater likelihood of prevalent mobility limitations and disability. This population of older men (mean age of 84.2 y) reported multiple chronic diseases. Men in the higher quartiles of D3Cr muscle mass had significantly lower prevalence of type 2 diabetes, myocardial infarction, chronic heart failure, and COPD. In addition, men in higher quartiles of D3Cr muscle mass/body mass generally had better cognitive function, had higher levels of physical activity, greater Life-Space, and had markedly lower levels of exhaustion and fatigue. In longitudinal analyses over 3-5 years of follow-up, low D3Cr muscle mass was also associated with increased risk of hip fractures, injurious falls, incident disability and mortality. Generally, the association of low D3Cr muscle mass with these outcomes was not explained by weakness or slow walking speed and there were few associations between low lean mass and these outcomes.

